# Optimal margins for early stage peripheral lung adenocarcinoma resection

**DOI:** 10.1186/s12885-021-08251-3

**Published:** 2021-05-11

**Authors:** Pan Yin, Bingqing Yue, Ji Zhang, Dong Liu, Dongyu Bai, Guang Zhao, Chutong Huang, Guojun Geng, Jie Jiang, Yongxiang Su, Xiuyi Yu, Jingyu Chen

**Affiliations:** 1 Wuxi Lung Transplant Center, Wuxi People’s Hospital Affiliated to Nanjing Medical University, 299 QingYang Road, Wuxi, 214023 China; 2 Department of Pathology, The First Affiliated Hospital of Xiamen University, 55 Zhenhai Road, Xiamen, 361003 China; 3 Department of Thoracic Surgery, The First Affiliated Hospital of Xiamen University, 55 Zhenhai Road, Xiamen, 361003 Fujian China

**Keywords:** Peripheral lung adenocarcinoma, Sublobar resection, Margin distance, Hematoxylin and eosin staining, Pathological outcomes

## Abstract

**Background:**

A pathologically confirmed negative margin is required when performing sublobar resection in patients with early stage peripheral lung adenocarcinoma. However, the optimal margin distance to ensure complete tumor resection while preserving healthy lung tissue remains unknown. We aimed to establish a reliable distance range for negative margins.

**Methods:**

A total of 52 intraoperative para-cancer tissue specimens from patients with peripheral lung adenocarcinoma with pathological tumors ≤2 cm in size were examined. Depending on the distance from the tumor edge (D), the para-cancer tissues were divided into the following five groups: D < 0.5 cm (group I); 0.5 cm ≤ D < 1.0 cm (group II); 1.0 cm ≤ D < 1.5 cm (group III); 1.5 cm ≤ D < 2.0 cm (group IV); and D ≥ 2.0 cm (group V). During pathological examination of the specimens under a microscope, the presence of atypical adenomatous hyperplasia or more severe lesions was considered unsafe, whereas the presence of normal lung tissue or benign hyperplasia was considered safe.

**Results:**

Group V, in which the margin was the farthest from the tumor edge, was the safest. There were significant safety differences in between groups I and V (χ^2^ = 26.217, *P* < 0.001). Significant safety differences also existed between groups II and V (χ^2^ = 9.420, *P* < 0.005). There were no significant safety differences between group III or IV and group V (*P* = 0.207; *P* = 0.610).

**Conclusions:**

We suggest that when performing sublobar resection in patients with early stage peripheral lung adenocarcinoma with pathological tumor sizes ≤2 cm, the resection margin distance should be ≥1 cm to ensure a negative margin.

## Background

Lung cancer has the highest morbidity and mortality rates among malignant tumors worldwide [1]. Since the National Lung Screening Trial approved the use of low-dose computed tomography for lung cancer screening, this method has become popular in the population and an increasing number of early stage non-small cell lung cancer (NSCLC) cases have been diagnosed [2]. Radical lobectomy is the gold standard for treating early stage lung cancer, although, with the refinement of lung cancer stage definition, sublobar resection has become gradually suitable for partial ≤2-cm lung adenocarcinoma [3–6]. In addition, sublobar resection is an important tool because it assists in the diagnosis of suspicious malignant pulmonary nodules. The margin status has been associated with local recurrence and a positive margin may present a greater risk for the prognosis of a patient with lung cancer [7–10]. However, removal of the excessive lung tissues may affect their postoperative lung function, especially in cases of elderly patients or in those with poor lung function before surgery. At present, we often use rapid frozen pathological examination to assess the margin status of the resection during the operation. In this study, we aimed to evaluate the distance range for negative margins by analyzing the pathology of para-carcinoma tissues at different distances from the tumor in patients with peripheral lung adenocarcinoma with pathological tumor sizes ≤2 cm and determine a critical negative margin distance.

## Methods

### Patient samples and study procedures

**Fig. 1:**
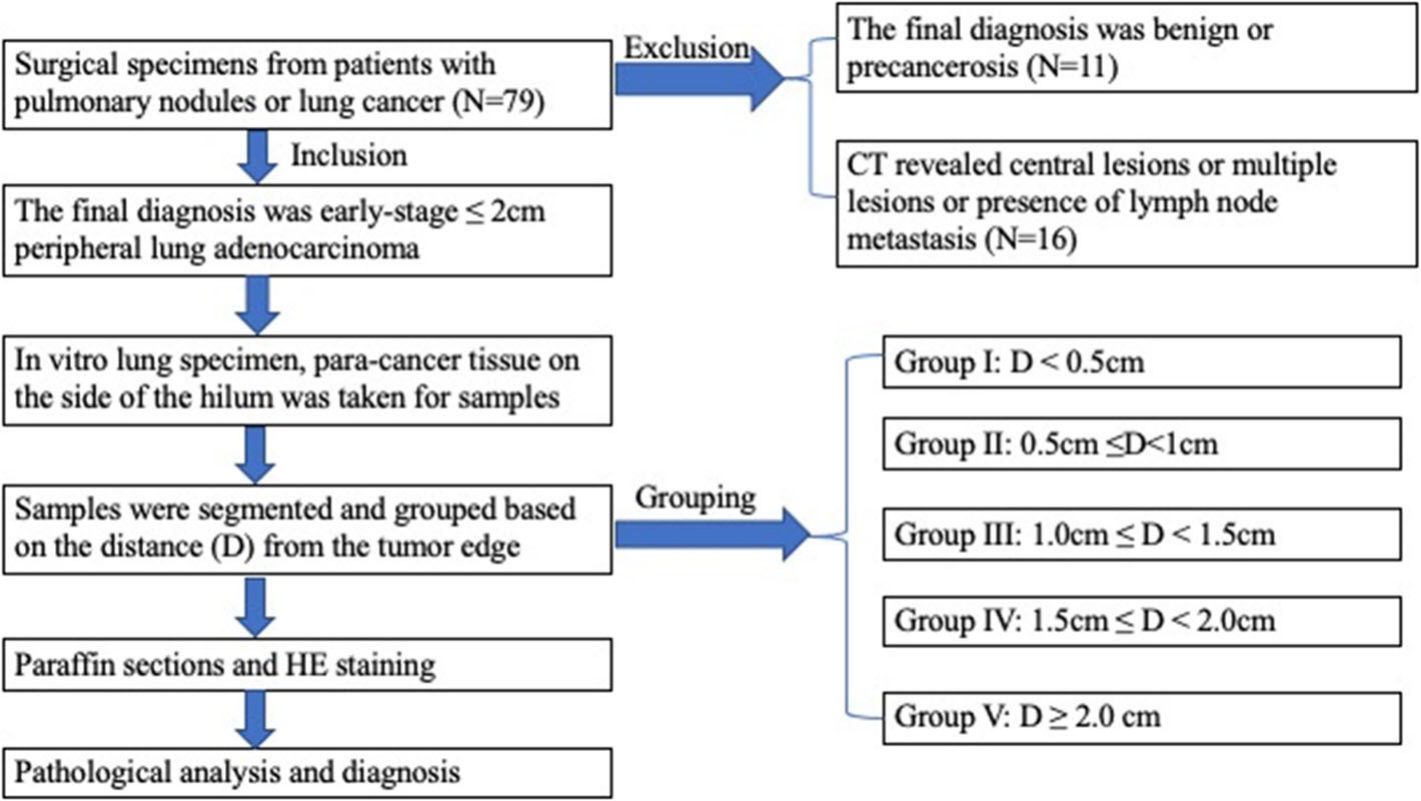
Flow chart of patient selection and grouping of para-cancer tissue specimens. N: number of patients; D: distance from the tumor edge

A total of 52 patients with early stage peripheral lung adenocarcinoma (pathological tumor size ≤2 cm) who underwent surgical treatment were included in the study. Surgical excision specimens were used to divide the para-carcinoma tissues on the side of the hilum into five groups depending on the distance from the tumor edge (D). The grouped para-carcinoma tissues were prepared into paraffin sections to observe the safety of the different distance groups. Statistical methods were used to identify the differences between the groups. The flowchart of patient selection and their division into groups are presented in Fig. 1. This study was performed in accordance with the Declaration of Helsinki and approved by the institutional review board of the First Affiliated Hospital of Xiamen University (No. REB2020#011). All patients consented to the surgery and provided written informed consent for study participation.

### Inclusion and exclusion criteria

The inclusion criteria were as follows: 1) patients provided informed consent before surgery and for the scientific research use of some surgical specimens; 2) imaging findings revealed peripheral and single lesions; 3) The pathological tumor size ≤2 cm without metastasis.

The exclusion criteria were as follows: 1) computed tomography revealed central lesions or multiple lesions; 2) final pathological diagnosis after the operation was benign; 3) final pathological examination was not lung adenocarcinoma, like small cell lung cancer or metastatic lung cancer.

### Sampling and grouping

#### Sampling

**Fig. 2:**
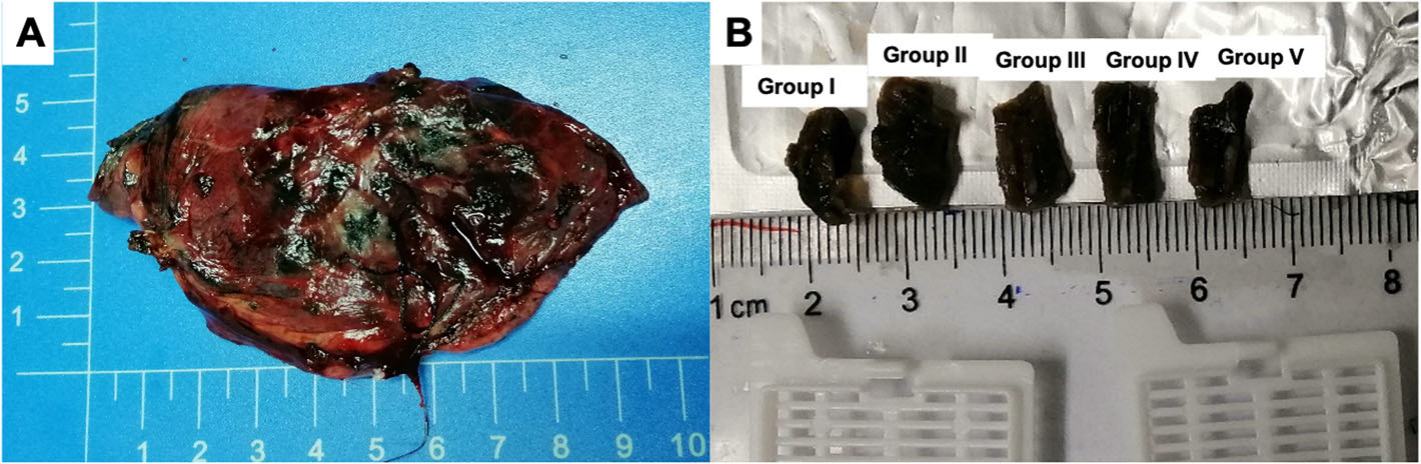
Images showing the sampling and grouping methods. **a** The lung specimen from the wedge resection is placed on the measuring table, and the para-cancer tissue between the tumor edge and cut-stapled line is evaluated. **b** Para-cancer tissues are divided into five groups according to the distance between the para-cancer tissues and the tumor edge, and the subdivided and grouped para-cancer tissues are placed into the embedding box for pathological examination

In the process of sampling and measurement, the specimens were in the state of lung collapse in vitro (Fig. 2a). The specimens are the paracancerous tissues on the hilar side and adjacent to the tumor nodules. We ensured that all para-cancer tissue samples were obtained without prejudice to the final clinicopathological diagnosis of the patient. On the side of the hilus pulmonis, the para-cancer tissue adjacent to the tumor was resected using a pathological knife. The side near the tumor was marked with a tissue-marker ink.

#### Grouping

According to the on the distance from the tumor edge, the para-cancer tissue specimens were divided into the following five groups: group I (D < 0.5 cm), group II (0.5 cm ≤ D < 1.0 cm), group III (1.0. ≤ D < 1.5 cm), group IV (1.5 cm ≤ D < 2.0 cm), and group V (D ≥ 2.0 cm). The grouped para-cancer tissues were placed into labeled embedding boxes and fixed with 10% formalin solution (Fig. 2b).

### Biopsy staining

The paraffin sections of all the grouped specimens were prepared using the procedures of fixation, dehydration, transparency, wax immersion, and embedding. We randomly selected six different positions in each distance group to make slices and then perform hematoxylin and eosin staining.

### Pathological criteria

**Fig. 3:**
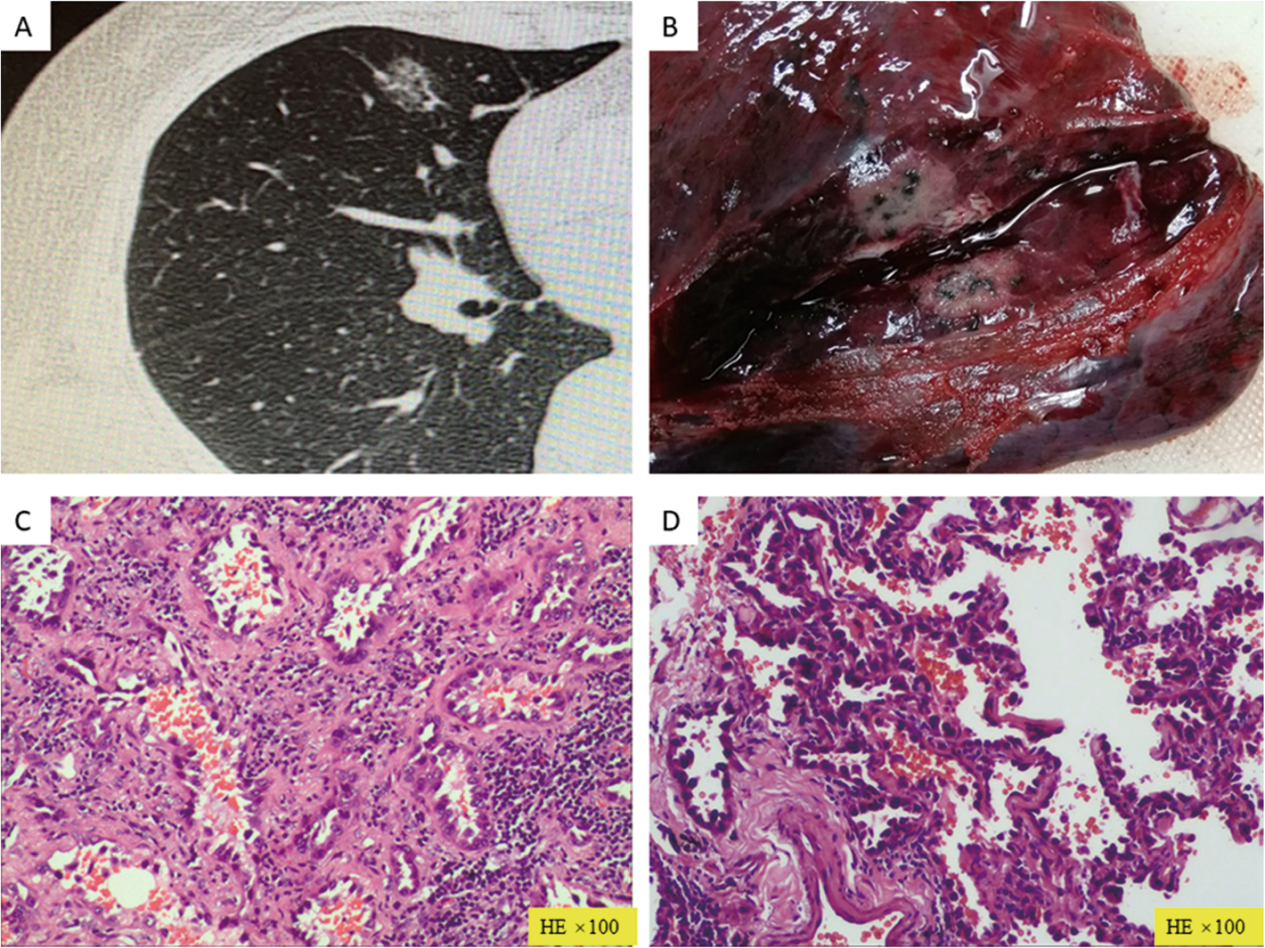
Typical case presentation. **a** Computed tomography image showing a mixed ground-glass nodule with a size of 1.7 cm in the middle lobe of the right lung. **b** A gray lesion is found in the isolated specimen of the right middle lobe. **c** IAC, as shown using postoperative pathology. **d** An AIS lesion is observed in para-carcinoma tissue 0.5–1.0 cm from the tumor margin. AIS: adenocarcinoma in situ; IAC: invasive adenocarcinoma

After performing hematoxylin and eosin staining, the paraffin sections were evaluated under a pathological microscope. A microscopic finding of atypical adenomatous hyperplasia (AAH) or of more severe, but not precancerous, lesions was considered unsafe (Fig. 3). A finding of benign hyperplasia or normal lung tissue was considered safe. When the remote group of the same case was considered to be unsafe, the nearby group was considered to be unsafe by default.

### Statistical analyses

The baseline and clinical outcome data (sex, age, lesion size, lesion location, lesion type, and final pathological results for each patient) were analyzed and recorded using descriptive statistics. The pathological results of the grouped para-cancer tissues were compared using the χ^2^ test. SPSS version 16 (SPSS Inc., Chicago, IL, USA) was used for all statistical analyses. *P*-values < 0.05 were considered indicative of statistical significance.

## Results

### Patient characteristics

Of the 52 patients who underwent surgical treatment for peripheral lung adenocarcinoma (pathological tumor size ≤2 cm), 20 were men and 32 were women (Table 1). Their ages ranged from 28 to 75 years, with an average age of 58 years; the mean tumor size was 1.4 ± 0.6 cm. Imaging revealed the following tumor types: solid pulmonary nodules in 13 patients (25%), subsolid nodules in 21 patients (40.4%), and ground-glass nodules in 18 patients (34.6%). The average consolidation/tumor ratio was 0.44 ± 0.43. The tumors were distributed in the upper lobe of the right lung in 17 patients (32.7%), middle lobe of the right lung in 3 patients (5.8%), inferior lobe of the right lung in 11 patients (21.1%), upper lobe of the left lung in 13 patients (25%), and inferior lobe of the left lung in 8 patients (15.4%). The surgical procedures were wedge resection in 20 patients (38.5%), segmentectomy in 4 patients (7.7%), and lobectomy in 28 patients (53.8%). According to the 2015 World Health Organization Classification of Lung Cancer [11], the final pathological results were adenocarcinoma in situ (AIS) in 3 patients (5.8%), minimally invasive adenocarcinoma (MIA) in 15 patients (28.8%), and invasive adenocarcinoma (IAC) in 34 patients (65.4%).

**Table 1:** General characteristics of our patients

Characteristics	Value
Total	52
Sex (male/female)	20/32
Age (years)	56 (28–75)
Pathological tumor size (cm)	1.4 ± 0.6
C/T radio	0.44 ± 0.43
smoking/non-smoking	13/39
Imaging type	
SPN (n, %)	13 (25)
SSN (n, %)	21 (40.4)
GGO (n, %)	18 (34.6)
Tumor Location	
Right upper (n, %)	17 (32.7)
Right middle (n, %)	3 (5.8)
Right inferior (n, %)	11 (21.1)
Left upper (n, %)	13 (25)
Left inferior (n, %)	8 (15.4)
Surgical type	
Wedge resection (n, %)	20 (38.5)
Segmentectomy (n, %)	4 (7.7)
Lobectomy (n, %)	28 (53.8)
Pathologic type	
AIS (n, %)	3 (5.8)
MIA (n, %)	15 (28.8)
IAC (n, %)	34 (65.4)

SPN, solid pulmonary nodules; SSN, subsolid nodules; GGO, ground-glass nodules; AIS, adenocarcinoma in situ; MIA, minimally invasive adenocarcinoma; IAC, invasive adenocarcinoma; C/T radio, consolidation/tumor ratio

### Pathological outcomes

A total of 260 pathological paraffin sections were prepared from 52 para-cancer specimens, which were grouped according to the D. Using pathological microscopic observations, it was found that in group I (D < 0.5 cm), 29 cases (55.8%) were safe and 23 (54.2%) were unsafe, including 6 cases being unsafe by default and 5, 7, 2, and 3 cases being unsafe owing to findings of AAH, AIS, MIA, and IAC, respectively. In group II (0.5 cm ≤ D < 1.0 cm), 41 cases (78.8%) were safe and 11 (21.2%) were unsafe, including 3 cases being unsafe by default and 4, 3, and 1 case(s) being unsafe due to findings of AAH, AIS, and MIA, respectively. In group III (1.0 cm ≤ D < 1.5 cm), 47 cases (90.4%) were safe and 5 (9.6%) were unsafe, including 3 cases being unsafe by default, 1 case owing to findings of AAH, and 1 case due to MIA. In group IV (1.5. ≤ D < 2.0 cm), 49 cases (94.2%) were safe and 3 (5.8%) were unsafe, including 1 case that was unsafe by default, 1 case owing to findings of AAH, and 1 case due to AIS. In group V (D ≥ 2.0 cm), 51 cases (98.1%) were safe and 1 (1.8%) was unsafe, which was revealed to be due to a finding of AAH (Table 2).

**Table 2:** Pathological results of the groups

Outcomes	Group I	Group II	Group III	Group IV	Group V
Safe	29 (55.8%)	41 (78.8%)	47 (90.4%)	49 (94.2%)	51 (98.1%)
Unsafe	23 (44.2%)	11 (21.2%)	5 (9.6)	3 (5.8%)	1 (1.9%)
Default ^a^	6	3	3	1	0
AAH	5	4	1	1	1
AIS	7	3	0	1	0
MIA	2	1	1	0	0
IAC	3	0	0	0	0

^a^ Default means that the remote group of the same case is unsafe, the close group is unsafe by default

### Critical negative distance

**Fig. 4:**
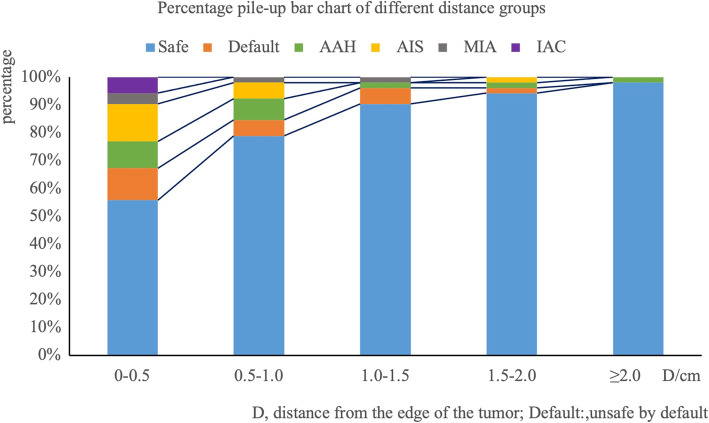
Percentage pile-up bar of different distance groups. As the distance from the edge of the tumor increases, the appearance of lesions decreases and the safety increases

According to the pathological results of each D-based group, the safety (negative proportion) rates from groups I to V were 55.8, 78.8, 90.4, 94.2, and 98.1%, respectively. The farther the para-cancer tissues were from the tumor, the safer they were (Fig. 4). Based on previous studies and current consensus, we defined group V (D ≥ 2.0 cm) as the safest group and set it as the control group [7, 8]. However, there was still a patient whose group V para-cancer tissues were identified as having AAH with no abnormality between groups II and IV. We suspected that the only AAH lesions in the group V para-cancer tissue of this patient might have been the primary lesions. Groups I to IV were compared with group V (control group), in which the margin was the farthest from the tumor edge. According to the χ^2^ test results, there was a significant difference in safety between groups I and V (χ^2^ = 26.217, *P* < 0.001). Group I (D < 0.5 cm) was significantly less safe than group V (D ≥ 2.0 cm). Group II (0.5 cm ≤ D < 1.0 cm) was also significantly less safe than group V (χ^2^ = 9.420, *P* = 0.002). There was no significant difference in safety when comparing group III (1.0 cm ≤ D < 1.5 cm) or group IV (1.5 cm ≤ D < 2.0 cm) with group V (*P* = 0.207 and 0.610, respectively) (Table 3).

**Table 3:** Safety comparison between the groups

			Safety evaluation		χ^2^	*p-*value ^b^
			Safe	Unsafe		
Groups	Group V ^a^ (control group)	count	51	1	–	–
		percentage	98.1%	1.9%		
	Group I	count	29	23	26.217	<0.001
		percentage	55.8%	44.2%		
	Group II	count	41	11	9.42	0.002
		percentage	78.8%	21.2%		
	Group III	count	47	5	1.592	0.207
		percentage	90.4%	9.6%		
	Group IV	count	49	3	0.260	0.610
		percentage	94.2%	5.8%		

^a^ Group V was set as the control group,

^b^ *p* < 0.05 indicated significance

## Discussion

Presently, sublobar resection is considered suitable for patients with early stage lung cancer with mainly ground-glass opacities or for those with poor cardiopulmonary function who are unable to tolerate radical lobectomy [4, 5]. It is still controversial whether the gold standard of radical lobectomy established by the Lung Cancer Study Group remains applicable for the treatment of early stage NSCLC (≤ 2 cm) [12]. Several recent randomized controlled trials have compared the efficacy of lobar and sublobar resection for early stage ≤2-cm NSCLC tumors [13–15]. Meanwhile, as an increasing number of pulmonary nodules are detected, sublobar resection has become an important diagnostic and therapeutic method for ≤2-cm suspected malignant pulmonary nodules [16]. Peripheral lung adenocarcinoma is the most common type of early stage NSCLC (≤ 2 cm).

To date, the surgical margin distance of sublobar resection for cases of early stage lung adenocarcinoma (≤ 2 cm) remains controversial [7–9]. Sublobar resection has been shown to have a higher local recurrence rate than lobectomy for T1N0 lung cancer, although the reasons for the higher local recurrence after sublobar resection have not been established [12]. Many studies have confirmed that the quality of the surgical margin is an important risk factor for local recurrence after sublobar resection [10, 17–19]. Nevertheless, pathological evaluation under a microscope remains still the main strategy for evaluating the margin quality. A sufficient margin distance is an important criterion for ensuring a negative margin (no residue under the microscope). In recent years, preoperative positioning technology for small lung tumors and pulmonary nodules has progressed greatly, thus, making it easier to determine the intraoperative margin distance of sublobar resection [20–23]. However, there are no reports on the margin distance that is required to obtain the most reliable negative margin in patients with peripheral lung adenocarcinoma with tumor sizes ≤2 cm who are undergoing sublobar resection. To the best of our knowledge, the present study is the first to use para-cancer histopathological analysis to investigate the relationship between the negative margin and margin distance during sublobar resection for ≤2-cm peripheral lung adenocarcinoma.

Our study revealed that the safety of para-cancer tissues in the two groups (groups I and II) with a D < 1 cm was significantly lower than that in group V (D ≥ 2 cm). There was no significant difference in the safety of para-cancer tissues between group III (1.0 cm ≤ D < 1.5 cm) or group IV (1.5 cm ≤ D < 2.0 cm) and group V (D ≥ 2 cm). This finding revealed that the surgical margin was unsafe when the margin distance was < 1 cm. When the margin distance was ≥1 cm, the safety of the surgical margin did not benefit from the margin distance. Therefore, 1.0 cm was considered the critical distance for obtaining a reliable negative margin for ≤2-cm peripheral lung adenocarcinoma.

A study examining the optimal resection margin distance during sublobar resection suggested a maximum value that was greater than or equal to the tumor size [24]. In our real-world setting, the farthest distance of para-cancer tissues was set at > 2 cm (group V). However, there was still a case that revealed an AAH lesion in the farthest distance group, with no abnormalities observed in the three groups with shorter distances (0.5 cm ≤ D < 2.0 cm). We considered the possibility of discontinuous lesions in the para-cancer tissue or a single lesion independent of the tumor. Further studies are needed to determine whether this distant lesion affects local recurrence.

There are few studies on the surgical margin in patients with early stage peripheral lung adenocarcinoma who are undergoing sublobar resection, and the opinions on the surgical margin distance are inconsistent. In a retrospective analysis, Mohiuddin et al. [8] evaluated local recurrence according to varying margin distances. When the margin distance was > 1.5 cm, the control of local recurrence did not benefit from the margin distance, which is slightly different from the critical value of 1 cm found in our study. This may be related to selection bias in some retrospective studies. El-Sherif et al. [7] also concluded that local recurrence with a margin distance < 1 cm was significantly higher than that with a margin distance ≥1 cm.

Although this study was conducted in strict accordance with the norms for the process of research and analysis, it had certain limitations. First, in terms of sampling, owing to the softness of lung tissues, the measurements and groupings of para-cancer tissues in vitro may be inaccurate. Second, this was a single-center study with a small sample size. Third, to reduce the possibility of synchronous lung cancer, all of our patients’ preoperative CT images were single nodules, the pathological types were the same as the primary tumor, and double or multiple nodules were eliminated when the patients were enrolled. Although the possibility of multiple primary tumors was very small, we have not found an effective way to distinguish the relationship between “unsafe” pathological findings, such as AAH or AIS and tumor spread through air space or synchronous lung cancer. In the future, large-scale research is needed to confirm our results.

## Conclusions

In conclusion, the farther the resection margin distance was from the tumor edge, the more likely it was to result in a reliable negative margin. When sublobar resection is performed in patients with peripheral lung adenocarcinoma with early stage tumor sizes ≤2 cm or in those with suspected malignant pulmonary nodules, the margin distance should be ≥1 cm to ensure a reliable negative margin. Thus, additional prospective studies on optimal sublobar resection margins, postoperative survival, and recurrence are needed. Furthermore, to determine the optimal margin distance, studies clarifying the relationship between the margin distance and postoperative survival are needed.

## Data Availability

The datasets used during the current study are available from the corresponding author on reasonable request.

## Abbreviations

AAH: Atypical adenomatous hyperplasia
AIS: Adenocarcinoma in situ
D: Distance from the tumor edge
IAC: Invasive adenocarcinoma
MIA: Microinvasive adenocarcinoma
NSCLC: Non-small cell lung cancer
